# Newborn infant parasympathetic evaluation (NIPE) as a predictor of hemodynamic response in children younger than 2 years under general anesthesia: an observational pilot study

**DOI:** 10.1186/s12871-019-0774-y

**Published:** 2019-06-11

**Authors:** Kan Zhang, Siyuan Wang, Lei Wu, Yun’an Song, Meihua Cai, Mazhong Zhang, Jijian Zheng

**Affiliations:** 1 Department of Anesthesiology, Shanghai Children’s Medical Center, Shanghai Jiao Tong University School of Medicine & National Children’s Medical Center (Shanghai), 1678 Dongfang Road, Shanghai, 200127 China; 2 Pediatric Clinical Pharmacology Laboratory, Shanghai Children’s Medical Center, Shanghai Jiao Tong University School of Medicine & National Children’s Medical Center (Shanghai), Shanghai, China; 3 Department of Anesthesiology, 3201 Hospital, Xi’an Jiaotong University Health Science Center, Hanzhong, Shaanxi Province China

**Keywords:** Newborn infant parasympathetic evaluation, Nociception, Analgesia, Pediatrics, Respiratory sinus arrythmia

## Abstract

**Background:**

It is still unknown whether newborn infant parasympathetic evaluation (NIPE), based on heart rate variability (HRV) as a reflection of parasympathetic nerve tone, can predict the hemodynamic response to a nociception stimulus in children less than 2 years old.

**Methods:**

Fifty-five children undergoing elective surgery were analyzed in this prospective observational study. Noninvasive mean blood pressure (MBP), heart rate (HR) and NIPE values were recorded just before and 1 min after general anesthesia with endotracheal intubation as well as skin incision. The predictive performance of NIPE was evaluated by receiver-operating characteristic (ROC) curve analysis. A significant hemodynamic response was defined by a > 20% increase in HR and/or MBP.

**Results:**

Endotracheal intubation and skin incision caused HR increases of 22.2% (95% confidence interval [CI] 17.5–26.9%) and 3.8% (2.1–5.5%), MBP increases of 18.2% (12.0–24.4%) and 10.6% (7.7–13.4%), and conversely, NIPE decreases of 9.9% (5.3–14.4%) and 5.6% (2.1–9.1%), respectively (all *P* < 0.01 vs. pre-event value). Positive hemodynamic responses were observed in 32 patients (62.7%) during tracheal intubation and 13 patients (23.6%) during skin incision. The area under the ROC curve values for the ability of NIPE to predict positive hemodynamic responses at endotracheal intubation and skin incision were 0.65 (0.50–0.78) and 0.58 (0.44–0.71), respectively.

**Conclusions:**

NIPE reflected nociceptive events as well as anesthestic induction in children less than 2 years undergoing general anaesthetia. Nevertheless, NIPE may not serve as a sensitive and specific predictor to changes in hemodynamics.

**Trial registration:**

This study was registered on May 3, 2018 in the Chinese Clinical Trail Registry; the registration number is (ChiCTR1800015973).

## Background

Endotracheal intubation and skin incision are two of the strongest noxious stimuli received by surgical patients under general anesthesia [1]. From one perspective, sufficient analgesic levels are critical to avoid unexpected movements, sympathetic reactions with consequent cardiovascular complications, and the development of pain memory. From another perspective, restriction to a minimum dosage of analgesic is desirable to avoid opioid-induced hyperalgesia, respiratory depression, and nausea as well as to achieve a shorter perioperative treatment period [2, 3]. Due to a lack of reliable tools for predicting and assessing the balance between analgesia and nociception during general anesthesia, clinicians mainly use classical symptoms of insufficient analgesia including increases in heart rate (HR), blood pressure, lacrimation, and sweating to tailor the administration of analgesic drugs, an approach that can reduce the side-effects of opioid overdosage but not underdosage [1, 4].

Subcortical-derived autonomic nervous system changes induced by nociceptive stimuli was shown to be reflective of the balance between nociception and analgesia [5–13]. Two parameters, the newborn infant parasympathetic evaluation (NIPE) and analgesia nociception index (ANI, MDoloris Medical Systems, Lille, France), were derived from a real-time reliable analysis of HR variability (HRV) in a time window of 64 s on a scale from 0 (maximum of nociception) to 100 (complete analgesia). NIPE is the neonatal version of the ANI used in adults [14, 15]. It has been shown that the autonomic nervous system responses to a noxious stimuli would change with the advancing age. As the nervous system matures, sympathetic HR modulation increases, while parasympathetic modulation decreases [16]. The ANI used in adult could not be adapted directly to children less than 2 years old due to the higher respiratory rate and heart rate in children [17]. The NIPE, on the other hand, reflects the parasympathetic tone. It was found that NIPE would decrease significantly in newborn infants after a painful surgical procedure [8]. The NIPE was also significantly reduced in babies borned by instrument-assisted delivery when compared to those delivered naturally [9].

In adult patients, the automomic index ANI had been used to predict hemodynamic changes associated with painful stimulation [10–13]. Study of the prediction ability of the NIPE in children has not yet been reported.

In this observational pilot study, two manuvors, namely endotracheal intubation and skin incision, were chosen as the noxious stimuli. As a primary endpoint, we evaluate whether the pre-event value of NIPE would be a good predictor of the hemodynamic responses of such stimulation.

## Methods

### Patients

This observational prospective study was approved by the ethics committee of Shanghai Children’s Medical Center affiliated to Shanghai Jiao Tong University (SCMCIRB-K2018049) prior to its start and was registerated in the Chinese Clinical Trial website ( http://www.chictr.org.cn/showproj.aspx?proj=27154, ChiCTR1800015973). Patients were enrolled over a 4-month period between June 2018 and September 2018. Full-term pediatric patients aged 1 month to 2 years with an American Society of Anesthesiologists physical status score I~II were included. Patients were scheduled for elective general or urinary surgery.

We excluded children who had a history of premature delivery or neurological, cardiac or respiratory conditions. Children who required prolonged resuscitation at birth, underwent general anaesthesia within the preceding week of study, experienced prolonged exposure to pain, and those who were currently receiving drugs with known effects on sympathetic and parasympathetic activity were also excluded. Written informed consents were obtained from the parents of study subjects.

### Anesthetic technique and monitoring

All pediatric patients were fasted according to the relevant guidelines [18]. Crystalloid fluid (Ringer’s acetate) containing 5% glucose was given in the ward by the attending surgeon or in the operating theater by the anesthesiologist in charge as appropriate. Oral midazolam 0.5 mg/kg as sedative premedication was administered to all children 30 min before patients were transferred to the operating room. All patients were accompanied by their parents or a senior nurse staff in our preparation room as they watched cartoon video or listened to stories for relaxation. Upon arrival in the operating theater, standard monitoring was applied using an anesthesia workstation (Datex-Ohmeda Aisys CS^2^, GE Healthcare, USA) with a three-lead electrocardiogram (ECG), pulse oximetry and non-invasive blood pressure measured at the arm. After an intraveous line was secured, all patients received Ringer’s acetate as maintenance fluid following the 4–2-1 rule.

Fentanyl 2–3 μg/kg was injected over a 15-s period. After 1 min, anesthesia was induced with propofol 2–3 mg/kg administered intravenously (i.v.) over 30 s. When the eyelash reflex was absent, the child was ventilated via a facemask with 100% oxygen. Rocuronium 0.6 mg/kg was administered i.v. for muscle relaxation, after adequate ventilation could be achieved via a facemask. A senior anesthesiologist (> 3 years experience) decided the timing of endotracheal intubation and performed intubation using video laryngoscope. The patients were then ventilated using pressure-controlled mode at a frequency of 20 breaths per minute (inhalation-to-exhalation ratio of 1:2). Peak inspiratory pressure would be adjusted to achieve tidal volume between 8 and 10 ml/kg and end-tidal carbon dioxide was maintained between 35 and 45 mmHg. Anesthesia was maintained with sevoflurane between 1.0–1.3 MAC according to the patient’s age and the fentanyl boluse administered, as clinically required.

### Study protocol

The MDoloris system (MDoloris Medical Systems, Loos, France) was integrated to the monitors of the anesthesia workstation for HRV analysis. After calibration, the instantaneous NIPE index was displayed on the monitor screen. The instantaneous NIPE was obtained based on four individual windows of 16 s. The R-R interval analysis for each 16-s block is based on a sliding window of 64 s. Continuous measurement of the indexes can be assumed by moving the 64-s window after each calculation. The sampling rate of the final parameters depends on the window moving period. In practice, a 1-s moving period is used [19].

The timing of endotracheal intubation was decided by the anesthesiologist who was blinded to the study protocol and the MDoloris monitoring system. The research team was responsible for recording the NIPE, HR and MBP measurements immediately before and 1 min after tracheal intubation and skin incision [20]. The changes in the MBP and HR during the observation were calculated by the following formula:

$$ \mathrm{change}\ \mathrm{percent}=\frac{{\left(\mathrm{HR}\ \mathrm{or}\ \mathrm{MBP}\right)}_{\mathrm{post}-\mathrm{stimulus}}-{\left(\mathrm{HR}\ \mathrm{or}\ \mathrm{MBP}\right)}_{\mathrm{pre}-\mathrm{stimulus}}}{{\left(\mathrm{HR}\ \mathrm{or}\ \mathrm{MBP}\right)}_{\mathrm{pre}-\mathrm{stimulus}}}\times 100\%. $$

A hemodynamic response was considered significant and clinically relevant if an increase in either parameter (HR and/or MBP) of more than 20% was observed after the noxious event. We also calculated the dynamic NIPE to examine its ability to predict a hemodynamic response [21].

$$ {\mathrm{NIPE}}_{\mathrm{dynamic}}=\frac{{\mathrm{NIPE}}_{\mathrm{post}-\mathrm{stimulus}}-{\mathrm{NIPE}}_{\mathrm{pre}-\mathrm{stimulus}}}{\left({\mathrm{NIPE}}_{\mathrm{post}-\mathrm{stimulus}}+{\mathrm{NIPE}}_{\mathrm{pre}-\mathrm{stimulus}}\right)/2}\times 100\% $$

### Statistical analysis

Patient data was presented as the mean (95% confidence interval [CI]) or median (interquartile range [IQR]) as appropriate. All data were tested for normal distribution using the Kolmogorov-Smirnov test. Variables before and after stimulation were compared using paired t tests. Receiver operating characteristic (ROC) curves and the associated area under curve (AUC) values were computed to assess the ability of the NIPE (pre-stimulation values) to predict hemodynamic reactivity. GraphPad Prism 7 (GraphPad Software, Inc., San Diego, CA, USA) and MedCalc version 18.2 for Windows (MedCalc Software, Ostend, Belgium) were used for statistical analysis. *P* values < 0.05 were considered statistically significant.

## Results

**Fig. 1:**
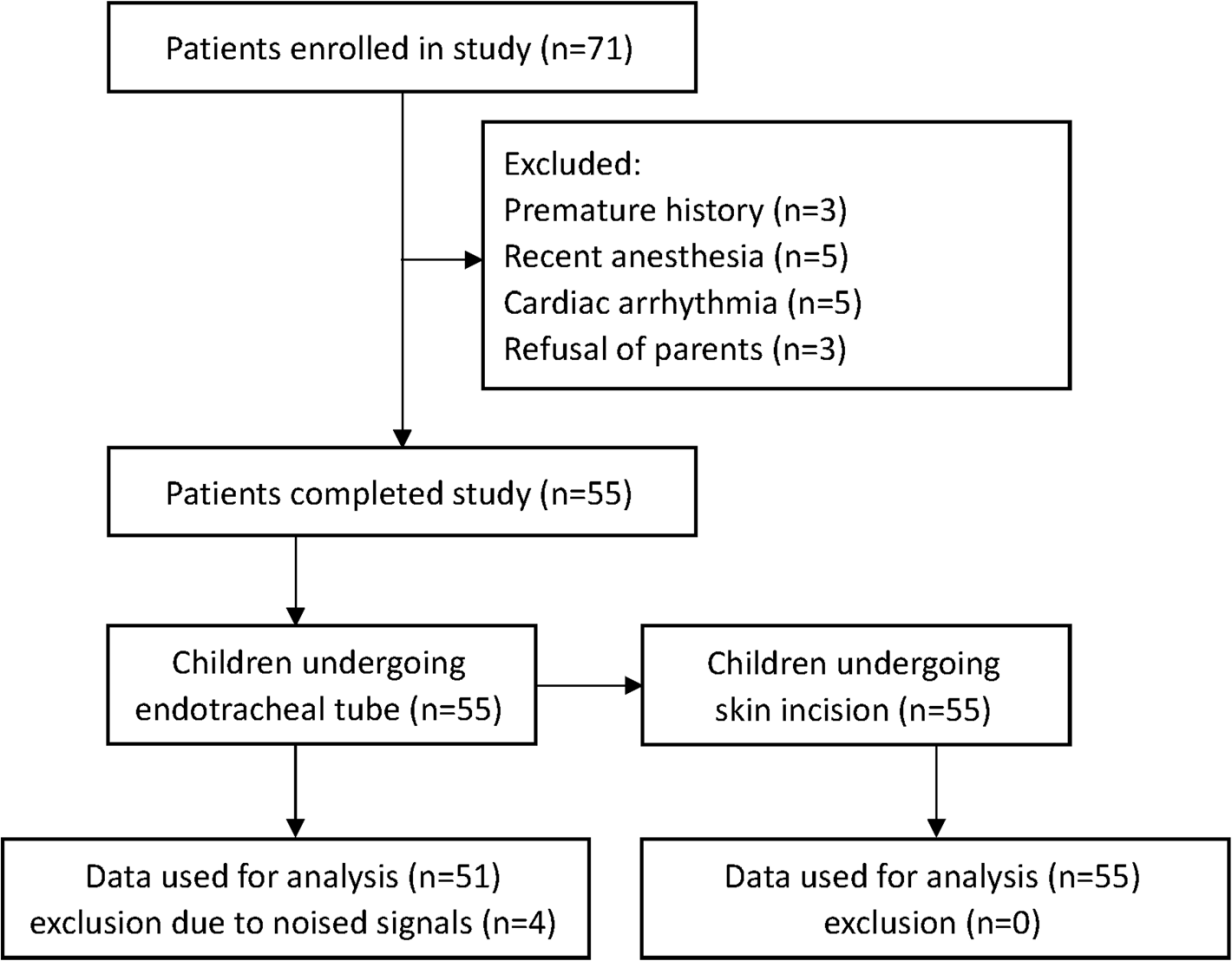
Flow digram of patient/data inclusion in this observational study

Seventy-one pediatric patients were initially recruited into this study. Of these, 16 patients were excluded due to a history of premature delivery, recent general anaesthesia, or arrythemia or a lack of parents’ permission for participation. Finally, 55 patients met the inclusion criteria and parents or informed consents were obtained. The characteristics of these patients are presented in Table 1. Of these pediatric patients, 47 children were male, and 8 were female. Their mean age was 1.3 years (95% CI 1.1–1.5 years), and their mean weight was 10.6 kg (10.0–11.1 kg). During the period of tracheal intubation, the NIPE recordings for four patients were complicated by noise due to poor electrode–skin contact. Thus, the final analysis included NIPE data from 51 patients undergoing endoutracheal intubation and 55 patients undergoing skin incision (Fig. 1).

**Table 1:** Detailed characteristics of pediatric patients

	Patients (*n* = 55)
Gender (M/F)	47/8
Age (years)	1.3 (1.1–1.5)
Weight (kg)	10.6 (10.0–11.1)
Height (cm)	76.1 (72.7–79.5)
ASA (I/II)	50/5
Surgery (n)	
General surgery	20/55
Urinary surgery	20/55
Orthopedic surgery	15/55
Propofol dose (mg/kg)	3.1 (2.8–3.5)
Fentanyl dose (μg/kg)	2.3 (2.1–2.6)

*ASA* American Society of Anesthesiologists physical status. Data are mean (95% CI) or absolute numbers

Induction of anaesthesia significantly changed the HR, MBP, and NIPE (all *P* < 0.01 vs. baseline). After induction of anaesthesia, in the absence of intubation, the HR and MBP decreased by 13% (95%CI 9–16%) and 15% (11–19%), respectively, while the NIPE increased by 19% (7–30%), as shown in Table 2.

**Table 2:** Change trends for mean blood pressure (MBP), heart rate (HR), and newborn infant parasympathetic evaluation (NIPE) at five time points

	T1	T2^#^	T3^*^	T4	T5^**^
MBP (mmHg)	73 ± 9	64 ± 11	75 ± 17	56 ± 9	62 ± 11
HR (bpm)	129 ± 23	111 ± 17	134 ± 17	111 ± 14	115 ± 15
NIPE	40 ± 12	45 ± 8	39 ± 7	60 ± 14	56 ± 13

^#^*P* < 0.01 T2 vs. T1; *P < 0.01 T3 vs. T2; **P < 0.01 T5 vs. T4. T1, before anesthesia induction; T2, immediately before intubation; T3, 1st min after intubation; T4, immediately before skin incision; T5, 1st min after skin incision

Data are shown as mean ± standard deviation

A positive hemodynamic response was observed during tracheal intubation in 32 patients (62.7%) and during skin incision in 13 patients (23.6%). Endotracheal intubation resulted in significant changes in the patients’ MBP, HR, and NIPE values (all *P* < 0.01 vs. before intubation). The average MBP and HR values increased by 18.2% (12.0–24.4%) and 22.2% (95%CI 17.5–26.9%), respectively, while the NIPE value decreased by 9.9% (5.3–14.4%). Skin incision also resulted in significant increases in the patients’ MBP [by 3.8% (2.1–5.5%)] an HR [by 10.6% (7.7–13.4%)] values and a decrease in the NIPE [by 5.6% (2.1–9.1%)] (all P < 0.01 vs. before incision). The MBP, HR, and NIPE values at each timepoint are presented in Table 2.

**Fig. 2:**
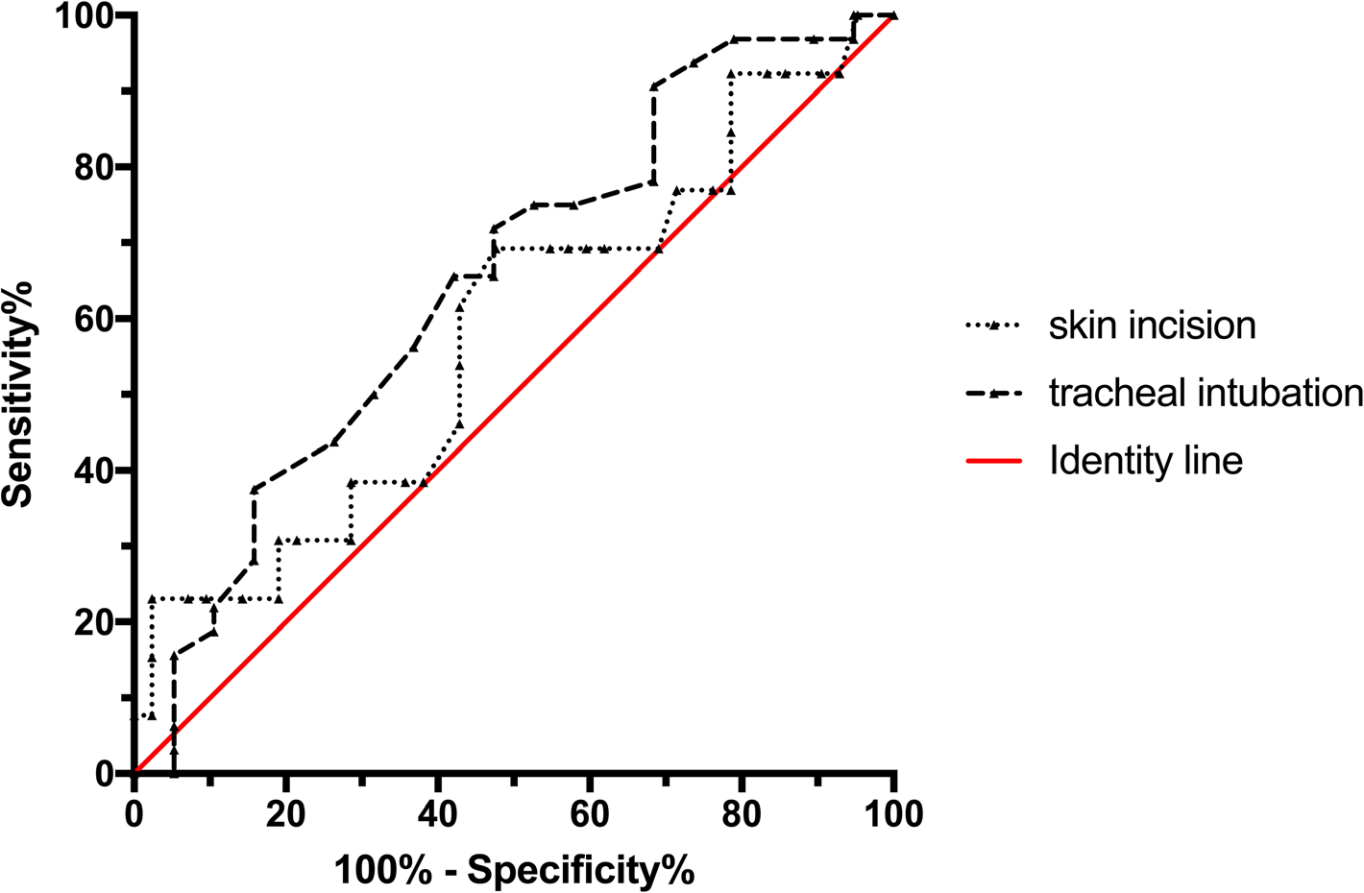
Predictive ability of NIPE for a traditional hemodynamic change at tracheal intubation and skin incision. The area under receiver operating characteristic curve (AUC) values for this predictive ability of NIPE were 0.65 (95%CI 0.50–0.78) at endotracheal intubation and 0.58 (0.44–0.71) at skin incision

The AUC values for the ability of NIPE to predict a positive hemodynamic response at endotracheal intubation and at skin incision were 0.65 (95%CI 0.50–0.78) and 0.58 (0.44–0.71), respectively. The best cut-off values (the optimal threshold) for the NIPE index at the respective events were 42 (sensitivity 71.9% and specificity 52.6%) and 60 (sensitivity 69.2% and specificity 52.4%). These results indicate the probability of correctly predicting a positive hemodynamic response based on the NIPE was similar to that achieved with a random coin toss (Fig. 2).

The AUC values for the ability of NIPE_dynamic_ to predict a positive hemodynamic response at endotracheal intubation and at skin incision were 0.68 (95%CI 0.53–0.80) and 0.54 (0.40–0.68), respectively. Thus, our results showed that the NIPE_dynamic_ also was not sufficiently sensitive and specific to predict a hemodynamic response to these events.

## Discussion

In the present study, we observed (1) the NIPE as well as NIPE_dynamic_ in children less than 2 years of age undergoing general anesthesia and found that these indexes failed to predict a hemodynamic response at tracheal intubation and skin incision. (2) The NIPE reflected nociceptive events as well as anesthestic induction in pediatric patients undergoing general anetshesia.

Several studies indicated ANI was able to predict movement and hemodynamic reactivity in adult patients [10–13]. Some studies observed hemodynamic response could not be anticipated based on the ANI [1, 21, 22]. Our study also did not find that the NIPE could predict a HR- or MBP-based response to nociceptive stimuli. The reason for this discrepancy of results are ultimately unclear. Patients in previous studies [10–13] did not receive neuromuscular blocking drugs before stimulation, whereas we used a regime with rocuronium. The previously described variation in autonomous stress response to tracheal intubation with and without neuromuscular blockade may partially explain the observed differences [23].

In consideration of perioperative safety, all children enrolled underwent ETT intubation after administration of a muscle relaxant. Children who were allowed to resume spontaneous breathing during surgery were not enrolled in order to avoid a statistical bias introduced by this anesthetic technique. The respiratory sinus arrythymia (RSA) was also affected by respiratory parameters (e.g., respiratory rate, tidal volume and inspiration-expiration ratio) [24, 25]. In the current study, the respiratory rate (RR) during mechanical ventilation was kept at a fixed number (20 breaths per minute), which was relatively lower than normal respiratory rate in awake children (~ 30 breaths per minute). Furthermore, in infants, the effects of different breathing rates with I:E ratios different from spontaneous breathing on HRV indices are unknown [26].

The pre-induction and pre-intubation NIPE values were low in our cohort (40 ± 12 and 45 ± 8, respectively). Absence of ventilation before intubation could explain the low NIPE values observed before intubation. NIPE is a measure for analyzing HRV that evaluates the parasympathetic activity by assessing RSA. Secondly, we speculate the a lower pre-intubation NIPE value may be associated with the injection pain and withdrawal response. Usually anesthesia for infants and children is induced with propofol and rocuronium. At last, HRV reflects the balance between parasympathetic and sympathetic nerve outflow from the central nervous system to the cardiac sinus node. Anxiety, which most likely plays a pathogenetic role, is associated with autonomic dysfunction [27]. Preoperative emotion (such as anxiety and fear) was shown to mediate RSA alteration in adults [28, 29]. Crying and struggling in older infants and toddlers may also lead to increased sympathetic nervous system activity. Acute stress, with subsequent release of catecholamines into the systemic circulation, causes abnormal HRV responses to acute pain [30]. In the pre-anesthesia period, although a series of strategies has been adopted, anxiety in children can be reduced but not eliminated, which also explains the low NIPE values that we observed prior to anesthesia induction.

Despite low pre-event values of NIPE in current study making a significant decline after noxious stimulus less likely, the NIPE showed a significanted change and reflected nociceptive events as well as anesthestic induction. Thus, NIPE may potentially aid the monitoring of nociception.

Finally, the present study has several limitations that should be noted. (1) NIPE evaluation requires ventilation, and NIPE analysis during apnea phases is questionable. (2) Under current sample size, weak response of hemodynamics to skin incision due to enough analgesia might have reduced the statistical power to detect prediction ability. A larger sample size is needed in further research.

## Conclusion

In conclusion, the NIPE reflected nociceptive events as well as anesthestic induction in infants and young toddlers undergoing general anaesthetia. Nevertheless, NIPE may not serve as a sensitive and specific predictor to changes in hemodynamics.

## Data Availability

The datasets used and/or analyzed during the current study are available from the corresponding author on reasonable request.

## Abbreviations

ANI: Analgesia nociception index
AUC: Area under curve
CI: Confidence interval
HR: Heart rate
MBP: Mean blood pressure
NIPE: Newborn infant parasympathetic evaluation
ROC: Receiver operating characteristic
RSA: Respiratory sinus arrythymia

## References

[1] Defresne A, Barvais L, Clement F, Bonhomme V (2018). Standardised noxious stimulation-guided individual adjustment of remifentanil target-controlled infusion to prevent haemodynamic responses to laryngoscopy and surgical incision: a randomised controlled trial. Eur J Anaesthesiol.

[2] Fechner J, Ihmsen H, Schuttler J, Jeleazcov C (2013). The impact of intra-operative sufentanil dosing on post-operative pain, hyperalgesia and morphine consumption after cardiac surgery. Eur J Pain.

[3] Fletcher D, Martinez V (2014). Opioid-induced hyperalgesia in patients after surgery: a systematic review and a meta-analysis. Br J Anaesth.

[4] Daccache G, Jeanne M, Fletcher D (2017). The analgesia nociception index: tailoring opioid administration. Anesth Analg.

[5] Boselli E, Bouvet L, Begou G, Dabouz R, Davidson J, Deloste JY, Rahali N, Zadam A, Allaouchiche B (2014). Prediction of immediate postoperative pain using the analgesia/nociception index: a prospective observational study. Br J Anaesth.

[6] Logier R, Jeanne M, De Jonckheere J, Dassonneville A, Delecroix M, Tavernier B (2010). PhysioDoloris: a monitoring device for analgesia / nociception balance evaluation using heart rate variability analysis. Conf Proc IEEE Eng Med Biol Soc.

[7] Boselli E, Jeanne M (2014). Analgesia/nociception index for the assessment of acute postoperative pain. Br J Anaesth.

[8] Faye PM, De Jonckheere J, Logier R, Kuissi E, Jeanne M, Rakza T, Storme L (2010). Newborn infant pain assessment using heart rate variability analysis. Clin J Pain.

[9] Rakza T, Butruille L, Thirel L, Houfflin-Debarge V, Logier R, Storme L, De Jonckheere J (2018). Short-term impact of assisted deliveries: evaluation based on behavioral pain scoring and heart rate variability. Clin J Pain.

[10] Boselli E, Bouvet L, Begou G, Torkmani S, Allaouchiche B (2015). Prediction of hemodynamic reactivity during total intravenous anesthesia for suspension laryngoscopy using analgesia/nociception index (ANI): a prospective observational study. Minerva Anestesiol.

[11] Funcke S, Sauerlaender S, Pinnschmidt HO, Saugel B, Bremer K, Reuter DA, Nitzschke R (2017). Validation of innovative techniques for monitoring nociception during general anesthesia: a clinical study using tetanic and Intracutaneous electrical stimulation. Anesthesiology.

[12] Gruenewald M, Herz J, Schoenherr T, Thee C, Steinfath M, Bein B (2015). Measurement of the nociceptive balance by analgesia nociception index and surgical Pleth index during sevoflurane-remifentanil anesthesia. Minerva Anestesiol.

[13] Jeanne M, Delecroix M, De Jonckheere J, Keribedj A, Logier R, Tavernier B (2014). Variations of the analgesia nociception index during propofol anesthesia for total knee replacement. Clin J Pain.

[14] Cremillieux C., Makhlouf A., Pichot V., Trombert B., Patural H. (2018). Objective assessment of induced acute pain in neonatology with the Newborn Infant Parasympathetic Evaluation index. European Journal of Pain.

[15] De Jonckheere J, Storme L. NIPE is related to parasympathetic activity. Is it also related to comfort? J Clin Monit Comput. 2019; 10.1007/s10877-019-00276-1. 10.1007/s10877-019-00276-1 30758689

[16] Oberlander TF, Grunau RE, Pitfield S, Whitfield MF, Saul JP (1999). The developmental character of cardiac autonomic responses to an acute noxious event in 4- and 8-month-old healthy infants. Pediatr Res.

[17] Constant I, Sabourdin N (2015). Monitoring depth of anesthesia: from consciousness to nociception. A window on subcortical brain activity. Paediatr Anaesth.

[18] Practice Guidelines for Preoperative Fasting and the Use of Pharmacologic Agents to Reduce the Risk of Pulmonary Aspiration (2017). Application to healthy patients undergoing elective procedures: an updated report by the American Society of Anesthesiologists Task Force on preoperative fasting and the use of pharmacologic agents to reduce the risk of pulmonary aspiration. Anesthesiology.

[19] De Jonckheere J, Rakza T, Logier R, Jeanne M, Jounwaz R, Storme L (2011). Heart rate variability analysis for newborn infants prolonged pain assessment. Conf Proc IEEE Eng Med Biol Soc.

[20] Xue FS, Liu KP, Liu Y, Xu YC, Liao X, Zhang GH, Li CW, Yang QY, Sun HT (2007). Assessment of small-dose fentanyl and sufentanil blunting the cardiovascular responses to laryngoscopy and intubation in children. Paediatr Anaesth.

[21] Boselli E, Logier R, Bouvet L, Allaouchiche B (2016). Prediction of hemodynamic reactivity using dynamic variations of analgesia/nociception index (ANI). J Clin Monit Comput.

[22] Ledowski T, Averhoff L, Tiong WS, Lee C (2014). Analgesia nociception index (ANI) to predict intraoperative haemodynamic changes: results of a pilot investigation. Acta Anaesthesiol Scand.

[23] Janda M, Bajorat J, Kudlik C, Pohl B, Schubert A, Noldge-Schomburg G, Hofmockel R (2013). Comparison of heart rate variability response in children undergoing elective endotracheal intubation with and without neuromuscular blockade: a randomized controlled trial. Paediatr Anaesth.

[24] Billman GE (2013). The effect of heart rate on the heart rate variability response to autonomic interventions. Front Physiol.

[25] Reyes del Paso GA, Langewitz W, Mulder LJ, van Roon A, Duschek S (2013). The utility of low frequency heart rate variability as an index of sympathetic cardiac tone: a review with emphasis on a reanalysis of previous studies. Psychophysiology.

[26] Lin IM, Tai LY, Fan SY (2014). Breathing at a rate of 5.5 breaths per minute with equal inhalation-to-exhalation ratio increases heart rate variability. Int J Psychophysiol.

[27] Miu AC, Heilman RM, Miclea M (2009). Reduced heart rate variability and vagal tone in anxiety: trait versus state, and the effects of autogenic training. Auton Neurosci.

[28] Sleigh JW, Henderson JD (1995). Heart rate variability and preoperative anxiety. Acta Anaesthesiol Scand.

[29] Ledowski T, Bein B, Hanss R, Tonner PH, Roller N, Scholz J (2005). Pseudocholinesterase activity increases and heart rate variability decreases with preoperative anxiety. Eur J Anaesthesiol.

[30] Terkelsen AJ, Molgaard H, Hansen J, Andersen OK, Jensen TS (2005). Acute pain increases heart rate: differential mechanisms during rest and mental stress. Auton Neurosci.

